# Diagnostic underestimation of atypical ductal hyperplasia and ductal carcinoma in situ at percutaneous core needle and vacuum-assisted biopsies of the breast in a Brazilian reference institution*

**DOI:** 10.1590/0100-3984.2014.0110

**Published:** 2016

**Authors:** Gustavo Machado Badan, Decio Roveda Júnior, Sebastião Piato, Eduardo de Faria Castro Fleury, Mário Sérgio Dantas Campos, Carlos Alberto Ferreira Pecci, Felipe Augusto Trocoli Ferreira, Camila D'Ávila

**Affiliations:** 1 PhD Fellow, Physician Assistant II at Unit of Imaging Diagnosis, Santa Casa de Misericórdia de São Paulo, São Paulo, SP, Brazil.; 2 PhD, Coordinator for the Sector of Breast Imaging, Santa Casa de Misericórdia de São Paulo, São Paulo, SP, Brazil.; 3 PhD, Full Professor at School of Medical Sciences, Santa Casa de São Paulo, Chief of Medical Practice at Santa Casa de Misericórdia de São Paulo, São Paulo, SP, Brazil.; 4 PhD, Physician Assistant II at Unit of Imaging Diagnosis, Santa Casa de Misericórdia de São Paulo, São Paulo, SP, Brazil.; 5 Imaging Diagnosis Specialists, Physician Assistants II at Unit of Imaging Diagnosis, Santa Casa de Misericórdia de São Paulo, São Paulo, SP, Brazil.; 6 Imaging Diagnosis Specialist, MD, Resident in General Radiology, Santa Casa de Misericórdia de São Paulo, São Paulo, SP, Brazil.

**Keywords:** Breast neoplasia, Core needle biopsy, Vacuum-assisted biopsy, Diagnostic techniques and procedures, Noninvasive intraductal carcinoma

## Abstract

**Objective:**

To determine the rates of diagnostic underestimation at stereotactic percutaneous core needle biopsies (CNB) and vacuum-assisted biopsies (VABB) of nonpalpable breast lesions, with histopathological results of atypical ductal hyperplasia (ADH) or ductal carcinoma in situ (DCIS) subsequently submitted to surgical excision. As a secondary objective, the frequency of ADH and DCIS was determined for the cases submitted to biopsy.

**Materials and Methods:**

Retrospective review of 40 cases with diagnosis of ADH or DCIS on the basis of biopsies performed between February 2011 and July 2013, subsequently submitted to surgery, whose histopathological reports were available in the internal information system. Biopsy results were compared with those observed at surgery and the underestimation rate was calculated by means of specific mathematical equations.

**Results:**

The underestimation rate at CNB was 50% for ADH and 28.57% for DCIS, and at VABB it was 25% for ADH and 14.28% for DCIS. ADH represented 10.25% of all cases undergoing biopsy, whereas DCIS accounted for 23.91%.

**Conclusion:**

The diagnostic underestimation rate at CNB is two times the rate at VABB. Certainty that the target has been achieved is not the sole determining factor for a reliable diagnosis. Removal of more than 50% of the target lesion should further reduce the risk of underestimation.

## Abstract (pt)

**Objetivo:**

Determinar o grau de subestimação diagnóstica de biópsias mamárias percutâneas estereotáxicas por agulha grossa (*core biopsy*) e assistidas a vácuo (mamotomia) em lesões não palpáveis, com resultados histopatológico de hiperplasia ductal atípica (HDA) ou carcinoma ductal in situ (CDIS) e que foram submetidas a exérese cirúrgica posteriormente. Como objetivo secundário, atribuiu-se a frequência de HDA e CDIS nos casos biopsiados.

**Materiais e Métodos:**

Foram revisados, retrospectivamente, 40 casos biopsiados com diagnóstico de HDA ou CDIS, entre fevereiro de 2011 e julho de 2013, e que posteriormente foram submetidos a cirurgia, cujo laudo histopatológico estava registrado no sistema interno de informações. Os resultados das biópsias foram comparados aos da cirurgia e a taxa de subestimação foi calculada de acordo com equações matemáticas específicas.

**Resultados:**

A taxa de subestimação diagnóstica da *core biopsy* foi 50% para HDA e 28,57% para CDIS, e da mamotomia foi 25% para HDA e 14,28% para CDIS. As HDAs representaram 10,25% do total de casos biopsiados, enquanto 23,91% foram CDIS.

**Conclusão:**

A taxa de subestimação diagnóstica é cerca de duas vezes maior na c*ore biopsy* em relação à mamotomia. A certeza do alvo atingido não é o único determinante para um diagnóstico preciso. Remover mais que 50% da lesão alvo poderá diminuir o risco de subestimação diagnóstica.

## INTRODUCTION

As demonstrated by large observational studies, the breast cancer mortality rate decreased by 31% over the last years, principally by the contribution from mammographic screening programs, which have led to early detection of the disease in a considerable number of cases^(1,2)^, emphasizing the relevance of imaging methods approached by several recent studies published in the Brazilian literature^(3-5)^.

Ductal carcinoma in situ (DCIS) is a precursor of invasive ductal carcinoma (IDC) and, previously to the introduction of mammography as a breast cancer screening method, it was rarely detected^(6)^, with an increase in its incidence from 2% to 20% in that period^(7,8)^ representing 15-20% of all breast cancers, besides representing 25-56% os all detected non palpable lesions^(8,9)^.

DCIS is characterized by proliferation of malignant ductal epithelial cells, with no noticeable sign of basal membrane invasion^(8)^, and the mammographic diagnosis is based on the presence of microcalcifications resulting from tissues necrosis and later calcification of debris and cellular secretion. Low-nuclear grade DCIS may remain silent for a long period or even remaining restricted to the ductus, while the high-grade ones show high growth rates, high mitotic indices, and almost always progress to high-grade invasive carcinoma^(10)^. Other radiological presentation forms of DCIS include nodules or architectural distortion^(8)^.

Atypical ductal hyperplasia (ADH) is considered to be the most common high-risk proliferative breast lesion for breast cancer^(11,12)^ and, because of the risk of diagnostic underestimation and likelihood of coexistence with DCIS and IDC, surgical resection is recommended after the histopathological diagnosis by means of percutaneous biopsy^(13)^. Histologically, it is defined as an abnormal ductal proliferation that might present with all or almost all DCIS characteristics, but affecting only a duct, and measuring < 2.0 mm in diameter^(11,14)^. According to the literature, it is diagnosed by 2-11% of percutaneous biopsies performed in breasts with suspicious mammographic findings^(13)^.

The options for early breast lesions diagnosis include core biopsy and vacuum-assisted breast biopsy (VABB) or surgical excisional biopsy. Both core biopsy and VABB represent alternatives to surgical excisional biopsy^(11,13,15,16)^, for their lower cost, lower morbidity, besides providing a more satisfactory aesthetic result. Such biopsies are outpatient procedures and do not require admission to a hospital to be performed^(15)^, allowing for immunohistochemical testing so as the surgeon is provided with appropriate information to guide the therapeutic decision making^(17,18)^.

In most cases, the biopsy results are in agreement with the post-surgical histopathological results, but there are cases of diagnostic underestimation characterized by detection of a less severe lesion at biopsy as compared with the histopathological findings at surgery^(17,18)^.

Although the theme has already been object of study in relevant international publications, the rates of diagnostic underestimation are quite variable in the literature. Additionally, few times have such rates been studied in the Brazilian population utilizing core biopsy^(15)^ and, as far as the authors are concerned, there are not any Brazilian study utilizing digital stereotactic biopsy system with a dedicated table with such a purpose. Therefore, this justifies the evaluation of core biopsy and VABB diagnostic underestimation rate in cases of ADH and DCIS later submitted to surgical resection in a Brazilian institution of reference, as well as associating it with the imaging features of breast lesions.

The present study was aimed at determining the rate of diagnostic underestimation at stereotactic core biopsy and VABB in cases of nonpalpable breast lesions classified as BI-RADS^®^ categories 3, 4 and 5, with histopathological results of ADH and DCIS later submitted to surgical resection in a Brazilian institution of reference in breast radiology. As a secondary objective, the frequency of such breast lesions in the biopsied cases was established.

## MATERIALS AND METHODS

Retrospective, analytical and cross-sectional study approved by the Committee for Ethics in Research, developed at the Unit of Imaging Diagnosis of Santa Casa de Misericórdia de São Paulo, São Paulo, SP, Brazil, evaluating histological results of 117 consecutive biopsies of patients in the age range between 37 and 84 years (mean: 52 years) with mammographic findings classified as BI-RADS categories 3, 4 and 5, referred to undergo stereotactic core biopsy or mammotomy in the period from February 1, 2011 to July 31, 2013.

For the selection of the study sample, the lesions were classified into three categories, namely, benign lesions, high-risk lesions, and malignant lesions. Exclusion criteria were the following: a) cases with benign histopathological results; b) cases with histopathological results of lesions at high risk for malignancy represented by complex sclerosing lesions and papilliferous lesions; c) cases with positive histopathological results of invasive cancer. The inclusion criteria were met by the biopsied cases with histopathological results of ADH and DCIS that constituted the present study sample.

All the biopsies were performed under digital stereotactic guidance (Lorad Multicare Platinum - Hologic; Bedford, USA) and performed by a medical team with at least 10 years of experience in breast imaging.

Core biopsies were performed with an automatic Magnum instrument (Bard; Covington, USA), with 2.2 cm penetration depth and coupled 12-gauge needle (SACN Biopsy Needle - Medical Device Technologies; Gainesville, USA), collecting 8 fragments. In the VABB procedures, 9-gauge needles were utilized (Suros System - Hologic; Bedford, USA), collecting 11 fragments. Both core biopsies and VABB procedures were performed under local anesthesia.

Because of the high cost of the needles, VABB was performed only in cases of suspicious clustered microcalcifications in areas of < 1.0 cm. The other cases of suspicious clustered microcalcifications, as well as nodules, focal asymmetry and architectural distortion were submitted to core biopsy.

All the biopsied fragments were submitted to radiography and considered to be satisfactory when the presence of microcalcifications was observed. Histopathological results were reported by pathologists with at least 10-year experience in breast diseases.

Diagnostic underestimation corresponded to those cases where biopsy histopathological results revealed ADH or DCIS and subsequent surgical resection demonstrated, respectively, histopathological results of DCIS and IDC.

The statistical analysis was descriptively performed and the rate of diagnostic underestimation was calculated by dividing the number of carcinomas in situ and/or invasive carcinomas at surgery by the number of ADH or DCIS, respectively, diagnosed at biopsy. The strength of association between the studied variables and diagnostic underestimation was analyzed by means of the respective confidence intervals (CI 95%). The exact Fisher's test was performed, with statistical significance set as *p* < 0.05. The frequency of ADH and DCIS in the present study was compared with that in the most relevant studies in the literature.

## RESULTS

In most of cases in the present study (80 cases - 68.3%) core biopsy was performed; and VABB procedures were performed in 37 (31.63%) cases.

Amongst the 117 cases submitted to interventional procedures, 70 (59.83%) presented benign histological results. Fifteen (12.81%) breast lesions were considered to be at high risk for malignancy, as follows: one (0.85%) case of papilliferous lesion; 2 (1.71%) cases of complex sclerosing lesion; and 12 (10.25%) cases of ADH. Also, 32 (27.35%) cases positive for malignancy, including 28 (23.91%) cases of DCIS and 4 (3.41%) cases of IDC. For the calculation of the diagnostic underestimation rate of breast biopsies, only 40 cases met the inclusion criteria - 28 with histological diagnosis of DCIS, and 12, of ADC.

Amongst the 12 cases of ADC, 8 (66.66%) were diagnosed by core biopsy and 4 revealed DCIS at the subsequent surgery, i.e., diagnostic underestimation in 50% of cases. The other 4 (33.33%) cases were diagnosed by VABB, and one demonstrated DCIS at total surgical resection, characterizing 25% of underestimation.

Amongst the 28 cases of DCIS, 14 (50%) were diagnosed by core biopsy, and 4 (28.57%) of them revealed to be IDC at the subsequent surgery. The other 14 (50%) cases were submitted to VABB and only 2 (14.28%) presented diagnostic underestimation, i.e., IDC at surgery.

According to the present study results, the mean diagnostic underestimation rate for core biopsy for ADH and DCIS in relation to histological results at subsequent surgery was 36% (CI 95%: 15-58), and for VABB, 16.7% (CI 95%: 0-36). Differences between core biopsy and VABB results were not statistically significant (exact Fisher's test; *p* > 0.1).

**Figure 1:**
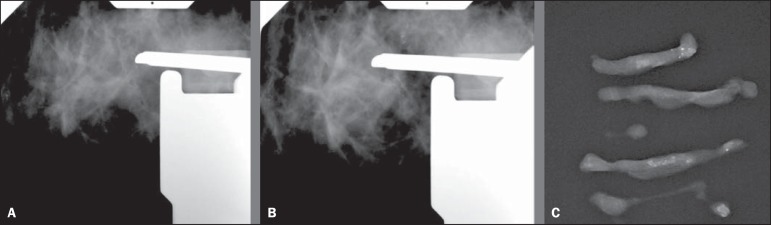
A 57-year-old patient with mammographic finding of amorphous and clustered microcalcifications (BI-RADS 4B). The patient was submitted to core biopsy, whose histological result was DCIS. At surgery, there was diagnostic underestimation (IDC). **A:** Pre-triggering stereotactic image showing the biopsy needle correctly directed toward the lesion. **B:** Post-triggering stereotactic image demonstrating the target transfixion by the needle. **C:** Radiography of the fragments identifying the presence of calcifications.

**Figure 2:**
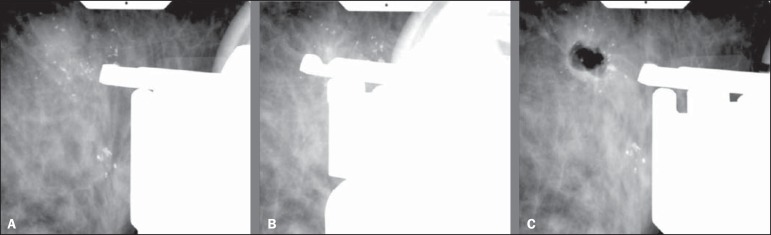
A 60-year-old patient with mammographic finding of gross, heterogeneous and clustered microcalcifications (BI-RADS 4B). VABB revealed AHD. At surgery, there was diagnostic underestimation (DCIS). **A:** Pre-triggering stereotactic image showing the biopsy needle correctly directed toward the lesion. **B:** Post-triggering stereotactic image demonstrating the needle in the target. **C:** Post-procedural stereotactic image revealing less than 50% of the removed target lesion.

One of the 4 cases with histological result of DCIS and diagnostic underestimation by core biopsy is represented on Figure 1. One of the 4 cases with diagnosis of ADH and diagnostic underestimation by VABB, with result of DCIS at subsequent surgery is represented on Figure 2.

In the present study, cases of diagnostic underestimation were not observed in relation to mammographic findings classified as BI-RADS categories 3 and 5. Amongst cases classified as BI-RADS category 4, the highest rate of diagnostic underestimation was observed in cases of clustered pleomorphic microcalcifications. Among the microcalcifications classified as category 4, with histological result of ADH at biopsy (10 cases), 5 (50%) were underestimated, while among the 24 cases with results of DCIS, 5 (20.83%) presented as IDC at the subsequence surgery. Among the 4 cases of architectural distortion with results of DCIS, there was one (25%) case of diagnostic underestimation (Table 1).

**Table 1:** BI-RADS categorization of mammographic findings, respective biopsy histological results and cases of diagnostic underestimation at subsequent surgery

	Breast biopsy histological results						
	Negative for malignancy			Positive for malignancy			
Mammographic findings according to BI-RADS categories	ADH	Other		DCIS	IDC	Diagnostic underestimation	Total number of cases
Category 3							
Clustered punctate microcalcifications	2	2		0	0	0	4
Focal asymmetry without associated findings	0	1		0	0	0	1
Category 4							
Clustered pleomorphic calcifications	10	59		24	0	10	93
Architectural distortion	0	9		4	0	1	13
Focal asymmetry with punctate microcalcifications	0	2		0	0	0	2
Category 5							
Segmental, pleomorphic, fine and branching microcalcifications	0	0		0	4	0	4
Total number of biopsies	12	73		28	4		117

In the study, among the 11 cases with diagnostic underestimation at subsequent surgery, a variation in lesion dimensions ranging from 0.8 cm to 2.6 cm (mean: 1.4 cm) was observed, and after the post-procedural mammographic images acquisition, a quantitative decrease of 10-50% (mean: 20%) in the number of microcalcifications was observed in cases of clustered pleomorphic microcalcifications and 20% in the case of architectural distortion.

## DISCUSSION

Breast cancer is the most frequent malignant tumor among women, after non-melanoma skin cancer. Early diagnosis is one of the main prognostic factors and the therapeutic approach will depend on the clinical staging and histopathological characteristics of the disease, on the clinical conditions, age and on the will of the patient^(19)^.

With the increase in the number of women submitted to annual mammographic screening, there is an enhancement of early detection of lesions^(15,20)^. According to the TNM classification by the American Joint Committee on Cancer, tumors staged as 0 (in situ) to IIB are considered to be initial breast tumors^(19)^.

Both core biopsy and VABB present results in agreement with the surgery in most cases, being considered to be the best tools in the diagnosis of breast lesions^(15-18,21)^. Additionally, they allow for planning the treatment in a single surgical time, including the axillary approach, avoiding unnecessary surgeries in up to 60% of cases and a second surgical time in 70% of cases^(15,18,21,22)^.

According data in the literature, the rate of diagnostic underestimation by core biopsy for DCIS ranges from 0% to 59%^(11,22)^, and in the present study was of 28.57%, while for ADH it ranges from 7% to 88%^(11,12,23)^, and in the present study was 50%.

The rate of diagnostic underestimation by VABB in the literature, for DCIS, ranges from 0% to 19%^(20)^, and in the present study was 14.28%, while for ADH it ranges from 20% to 56%^(11,12,14)^, and in the present study was 25%. According to Liberman et al., diagnostic underestimation occurs because many times there is coexistence of ADH, DCIS and IDC in a single lesion, and fragments of only ADH and DCIS might be collected at biopsy. Such authors have published in the literature that in about 66% of cases of diagnostic underestimation of ADH, the histopathological result at surgery is DCIS, and in 14-45% it may be IDC^(14)^. Those data suggest that breast biopsy with histological result of ADH require surgery for a more accurate diagnostic evaluation. Thus, all the 12 cases in the present study were submitted to surgical resection of the lesion.

VABB presents lower rates of diagnostic underestimation than core biopsy, a fact that is demonstrated by both the present study and the literature^(24,25)^. This is due to the fact that VABB provides larger, contiguous fragments and, consequently, more complete samples of the lesion, reducing the chances of false-negative results or underestimation^(26)^. In the present study, the mean rate of diagnostic underestimation for cases of ADH and DCIS was 16,7% (CI 95%: 0-36), that is lower as compared with core biopsy, whose underestimation rate was 36% (CI 95%: 15-58). But, because of the small size of the sample, there was no statistical significance (*p* = 0.1).

The results reported in the literature suggest that the diagnostic underestimation might be a result from inappropriate sampling ^(27)^. In the present study, 8 fragments were collected from the lesions submitted to core biopsy, and 11 at VABB, following the recommendations of some studies to collect at least 5 fragments at core biopsy, and more than 10 fragments at VABB, in order to increase the methods accuracy^(23,28-30)^. In one of their studies, Jackman et al. have reported an increase in the rate of diagnostic underestimation in breast lesions as less than 10 fragments were collected at VABB utilizing 11-gauge needles^(31)^. Also, the presence of calcifications was evaluated at radiography of the fragments in all the cases of microcalcifications to assure that the target had been reached. In the present study, no new biopsy was necessary. Such a result is attributed to the study method with the use of core biopsy in lesions > 1.0 cm, and to the experience of the team.

In the present study, 11 cases of diagnostic underestimation were found, 10 of them occurring in clusters of microcalcifications and one represented by architectural distortion. The authors observed that in all of such cases, less than 50% of the lesion was removed. According to Hoang et al. and, in agreement with the present study, "clustered microcalcifications" was the mammographic finding most associated with the chance of diagnostic underestimation, due to the difficulty to obtain representative samples of the entire lesion^(32)^. Several studies have demonstrated that diagnostic underestimation is less common in cases where the target-lesion is almost completely removed^(28)^. It is believed that, although the target has been reached, a higher number of samples correctly directed to the target, removing a greatest part of the lesion, could reduce the rate of diagnostic underestimation^(33)^.

However, it is important to highlight that the presence of a post-biopsy residual lesion is useful as a natural marker of the biopsied target and will serve as a guide in the case of future surgical approach, or even for the purpose of comparative analysis in mammographic follow-up.

Amongst the 117 biopsied cases, 12 (10.25%) ADH and 28 (23.91%) DCIS were found. Darling et al., in a study involving a large sample, have found a frequency of 16.7% of DCIS at percutaneous breast biopsies indicated for microcalcifications^(34)^. According to the literature, the rate of ADH diagnosed at biopsy ranged from 2% to 11%^(13,30,35)^. In a relevant meta analysis, an ADH frequency of 5% was observed at VABB with 11-gauge needles^(35)^. The authors attribute their results of ADH frequency within the expected superior limits and of DCIS above the ones reported in the literature to the small number of cases and also to the fact that 68.37% of the lesions were submitted to core biopsy, thus enhancing the chances of diagnostic underestimation.

Some limitations of the present study deserve to be mentioned. First, the number of cases of underestimation is not sufficient to support an accurate conclusion in a Brazilian population. For this reason, the findings of the present study should be taken as a preliminary result, despite the similarity between the present data and the ones reported in the international literature. Second, the study method itself, by restricting the use of VABB to cases of microcalcifications occupying an area < 1 cm, may have created a favorable bias for VABB, as it allows for resection of a greater part of the lesion, possibly reducing the diagnostic underestimation by the sample selection. On the other hand, as already discussed, microcalcifications are more closely related to the chance of diagnostic underestimation, which might enhance its rate. Although this can be considered as a limitation, it depicts the conditions of the daily clinical practice in a Brazilian public institution.

The authors consider the establishment of a rate of diagnostic underestimation of percutaneous stereotactic breast biopsy in a Brazilian service of reference as a small contribution of the present study.

## CONCLUSION

The authors observed that the rate of diagnostic underestimation is about two times higher in core biopsy as compared with VABB. This result corroborates data in the literature. VABB is a procedure that provides larger and contiguous fragments, which allows for a more accurate evaluation of the lesion, determining a reduction of the rate of diagnostic underestimation of ADH and DCIS in breast lesions.

Diagnostic underestimation might be associated with inappropriate sampling of the collected material because of the poor representativeness of the lesion. Certainty that the target has been achieved is not the sole determining factor for a reliable diagnosis. Resection of more than 50% of the target lesion should further reduce the risk of underestimation.
